# Binding Affinity and Interaction Profiles of Erinacines and Erinacerins with iNOS and NF-*κ*B Revealed by Molecular Dynamics Simulations

**DOI:** 10.3390/ijms27073145

**Published:** 2026-03-30

**Authors:** April Michelle Hernandez-Munguia, Andrés Reyes-Chaparro, Tomas Alejandro Fregoso-Aguilar, Aldo Yair Tenorio-Barajas, Jorge Alberto Mendoza-Pérez, Ricardo Aguilar-Garay, Dulce E. Nicolás-Álvarez

**Affiliations:** 1 Laboratorio de Hormonas y Conducta, Departamento de Fisiología, Escuela Nacional de Ciencias Biológicas, Instituto Politécnico Nacional, Ciudad de México 07700, Mexico; aprilmunguia0203@gmail.com (A.M.H.-M.); tfregoso@ipn.mx (T.A.F.-A.); 2 Departamento de Morfología, Escuela Nacional de Ciencias Biológicas, Instituto Politécnico Nacional, Ciudad de México 11340, Mexico; areyesch@ipn.mx; 3 Laboratorio de Nanobiotecnologia, Facultad de Ciencias Físico Matemáticas, Benemerita Universidad de Puebla, Av. San Cladio y 18 Sur, Col. San Manuel, Edif. FM6-108, Ciudad Universitaria, Puebla 72570, Mexico; aldo.tenorio@fcfm.buap.mx; 4 Laboratorio de Tecnologías Limpias, Desarrollo de Procesos Ambientales e Ingeniería Verde, Departamento de Ingeniería en Sistemas Ambientales, Escuela Nacional de Ciencias Biológicas, Instituto Politécnico Nacional, Ciudad de México 07700, Mexico; jmendozap@ipn.mx

**Keywords:** *Hericium erinaceus*, erinacines, erinacerins, molecular docking, molecular dynamics, MM-PBSA, iNOS, NF-*κ*B, neuroinflammation, binding affinity

## Abstract

Chronic neuroinflammation driven by microglial activation is a pathological hallmark of neurodegenerative diseases, and the NF-κB/iNOS signaling axis plays a central role in propagating this damage. NF-κB-mediated iNOS transcription generates excessive nitric oxide, causing oxidative neuronal injury. The medicinal mushroom *Hericium erinaceus* produces cyathane diterpenoid erinacines and isoindolinone erinacerins, both reported to attenuate neuroinflammation; however, the molecular basis of their interactions with iNOS and NF-κB remains poorly characterized. We screened 21 erinacerins and 18 erinacines against both targets using validated molecular docking, then subjected top-ranked candidates and negative controls to 100 ns molecular dynamics simulations, MM-PBSA binding free energy calculations (±SEM), per-residue energy decomposition, backbone RMSD, and ligand–protein minimum distance analyses, with quercetin as reference. The analysis revealed scaffold-dependent target selectivity: erinacerins exhibited preferential stability with iNOS (erinacerin L: RMSD 0.185 nm), whereas erinacines formed more stable complexes with NF-κB (erinacines G and J: RMSD < 0.36 nm). Minimum-distance monitoring confirmed that the elevated ligand RMSD in iNOS predominantly reflected surface relocation rather than dissociation. Erinacine S emerged as the most promising dual-target candidate ($\Delta G_{bind}$: −24.31 ± 0.16 and −14.24 ± 0.11 kcal/mol for iNOS and NF-κB, respectively), over twofold stronger than quercetin for iNOS. Negative controls revealed that docking-based ranking was target-dependent in its discriminative capacity, underscoring the need for MD-based refinement. These results identify erinacine S as a priority candidate for experimental validation.

## 1. Introduction

Neurodegenerative diseases are among the most pressing health challenges of the 21st century. In the context of global population aging, disorders such as Alzheimer’ s disease and Parkinson’ s disease affect millions of individuals worldwide and impose substantial burdens on patients, caregivers, and healthcare systems [1,2,3,4]. Although these conditions differ markedly in their clinical manifestations and primary pathological substrates, a prominent and widely recognized hallmark across major neurodegenerative disorders is chronic neuroinflammation sustained by persistent activation of microglia, the principal immune cells of the central nervous system [5,6]. When microglia remain activated for prolonged periods, continuous production of pro-inflammatory mediators—including tumor necrosis factor alpha (TNF-α), interleukin-1 beta (IL-1β), and interleukin-6 (IL-6)—together with reactive oxygen and nitrogen species, can damage surrounding neurons and contribute to a self-reinforcing cycle of inflammation and neurodegeneration [7,8]. A central signaling axis in this cascade involves nuclear factor kappa-light-chain-enhancer of activated B cells (NF-κB) and inducible nitric oxide synthase (iNOS): upon activation by pro-inflammatory stimuli, NF-κB translocates to the nucleus and drives iNOS transcription, leading to sustained nitric oxide (NO) production that, at elevated concentrations, generates reactive nitrogen species capable of oxidative neuronal injury [9,10]. Because NF-κB activation is the upstream event triggering iNOS expression, simultaneous modulation of both nodes has been proposed as a potentially advantageous strategy for attenuating neuroinflammatory damage [11].

Natural products have long served as a cornerstone of drug discovery and continue to provide high-value chemical scaffolds for therapeutic development. In fact, comprehensive analyses of approved medicines indicate that a substantial fraction of small-molecule drugs introduced over the past four decades are natural products, natural-product derivatives, or natural-product-inspired compounds [12]. Among the organisms currently under investigation, *Hericium erinaceus*—commonly known as the lion’ s mane mushroom—has attracted particular attention for its documented neuroprotective properties [13,14]. Long valued in traditional Asian medicine, this edible fungus produces two classes of bioactive secondary metabolites: Erinacines (ENCs), cyathane diterpenoids bearing a fused 5-6-7 tricyclic ring system, mainly in the mycelium [15], and Erinacerins (ENCNs), isoindolinone derivatives featuring a bicyclic lactam core, present in both mycelium and fruiting bodies [16,17]. Recognizing these distinct sources underscores the natural complexity of this organism’s chemical profile.

Several ENCs, notably ENC A, C, and S, have demonstrated the capacity to stimulate neurotrophic signaling and exert protective effects in experimental models relevant to ischemia, Parkinson’ s disease, depression, and neuroinflammation [18,19,20,21]. Among these, ENC A is the most extensively characterized member: it reduces iNOS expression and attenuates nitrotyrosine formation via p38 MAPK inactivation in ischemic injury models [22] and prevents LPS-induced iNOS upregulation in BV2 microglia while suppressing NF-κB phosphorylation [20]. Although ENC A was not carried forward to MD simulations in this study—as it was not among the top-ranked candidates for either target in the docking screening—its well-documented dual modulation of iNOS and NF-κB provides an important experimental reference point for interpreting the computational results obtained with structurally related ENCs. Importantly, while the in silico absorption, distribution, metabolism, and excretion (ADME) profiling reported in this manuscript predicts limited blood–brain barrier (BBB) permeability for most of the screened structures, experimental pharmacokinetic and distribution studies support CNS exposure at least for specific erinacines, including the detection of erinacine A in brain tissue after oral administration [23] and evidence consistent with BBB penetration for erinacine S [24]. Together, these data suggest that BBB permeability may be compound-dependent and may be influenced by formulation and metabolism, underscoring the value of integrating in silico predictions with experimental bioavailability date when interpreting neuroprotective outcomes.

Experimental evidence supports the anti-inflammatory potential of *H. erinaceus*–derived metabolites and indicates convergence on the NF-κB/iNOS axis. In microglial inflammatory models, erinacine A has been reported to reduce iNOS expression and attenuate inflammatory signaling associated with NF-κB activation under LPS-based stimulation paradigms [20,22], whereas erinacine C decreases nitric oxide output in BV2 microglia concomitantly with down-regulation of NF-κB signaling [21]. Collectively, these findings are consistent with a coordinated modulation of two mechanistically linked inflammatory nodes; however, the structural determinants that govern the recognition of these metabolites by iNOS and NF-κB remain insufficiently characterized. In this context, Wei et al. [25] reported a computational assessment of selected *H. erinaceus* diterpenoids against iNOS using molecular docking and molecular dynamics, employing quercetin as a benchmark ligand based on its reported ability to suppress inflammatory pathways involving iNOS and NF-κB [26,27]. Notably, that study focused on a limited subset of erinacines and did not include NF-κB as a target or extend the analysis to isoindolinone-based ENCNs. Therefore, an integrated and systematic comparison of ENCs and ENCNs across both iNOS and NF-κB remains limited in the available literature.

Computational methods, including molecular docking, molecular dynamics (MD) simulations, and binding free energy calculations, have become key tools in modern drug discovery by enabling rapid screening of chemical space, molecular-level hypothesis generation, and prioritization of candidates for experimental validation [28,29,30]. These approaches are particularly valuable in natural product research, where high structural diversity and variable physicochemical properties present both opportunities and challenges for systematic evaluation.

The present study was designed to address this knowledge gap by evaluating cyathane diterpenoids (ENCs) and isoindolinone derivatives (ENCNs) from *H. erinaceus* as candidate ligands for the mechanistically linked inflammatory targets iNOS and NF-κB. We employed an integrated in silico workflow comprising molecular docking (validated by redocking against reference ligands) for initial screening, 100 ns MD simulations to assess complex stability—monitored through ligand and backbone RMSD, RMSF, and ligand–protein minimum distance analyses—and MM–PBSA calculations to estimate relative binding free energies, complemented by per-residue energy decomposition. To evaluate the discriminative power of the docking-based selection, a subset of low-scoring compounds was additionally subjected to the full MD/MM-PBSA pipeline as negative controls. Quercetin was included as a positive-control reference to benchmark the computational protocol and facilitate comparison with prior computational work on *H. erinaceus* metabolites [25]. Importantly, its inclusion is also supported by experimental literature showing that quercetin can suppress inflammatory signaling relevant to the NF-κB/iNOS axis, including inhibition of LPS-induced nitrite production and downregulation of iNOS expression in macrophage models [26,31]. We further hypothesized that scaffold-level differences between ENCs (cyathane framework) and ENCNs (isoindolinone core) would translate into distinct interaction patterns and target preferences toward iNOS and NF-κB, providing mechanistic insights to guide future experimental investigations.

## 2. Results

### 2.1. Selected Compounds

Based on the docking screening, seven ENCNs and seven ENCs were selected for MD simulations. Table 1 and Table 2 present the 2D structures, PubChem identifiers, and molecular formulae of the selected compounds. Physicochemical properties, including molecular weights, are reported in Table 3.

Table 3 summarizes the physicochemical properties, drug-likeness compliance, pharmacokinetic predictions, and in silico toxicity assessment for all selected compounds. Among the ENCNs, erinacerin A and Q exhibited the highest lipophilicity (cLogP ≈ 4.9), while all ENCs showed favorable aqueous solubility and high predicted GI absorption. None of the selected compounds showed predicted mutagenic, tumorigenic, or reproductive toxicity. Notably, BBB permeability was predicted only for erinacerin A, whereas all ENCs were predicted as non-BBB-permeant under the SwissADME model—although experimental evidence suggests compound-dependent CNS exposure for specific erinacines, as discussed in the Introduction.

### 2.2. Molecular Docking

**Figure 1:**
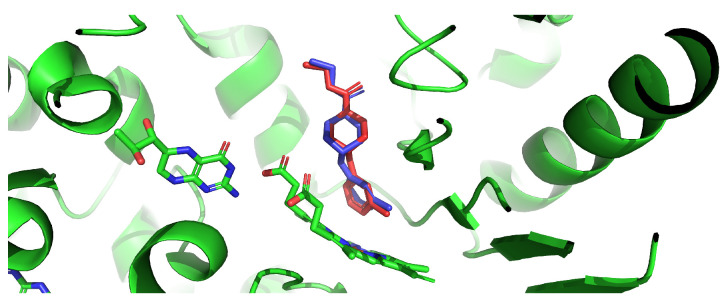
Redocking validation for iNOS. Superposition of the co-crystallized AR-C124798 (blue) and the redocked pose (red). Heavy-atom RMSD = 0.456 Å.

Prior to the screening campaign, the reliability of the docking protocol was assessed through two complementary tests. First, the co-crystallized ligand AR-C124798 (AT2) was extracted from the iNOS structure (PDB: 3E7G) and redocked into the receptor under blind docking conditions. Among the ten generated poses, one reproduced the crystallographic binding mode with a heavy-atom RMSD of 0.456 Å, well below the 2.0 Å threshold considered indicative of successful pose prediction [32] (Figure 1). The top-ranked pose (model 1, −8.3 kcal/mol) was located at an alternative site on the protein surface, which is expected under a blind docking protocol with a large search volume (>27,000 Å^3^) that intentionally explores the entire receptor without spatial bias. The successful reproduction of the native binding mode confirms that AutoDock Vina can identify the correct pose within the ensemble of predicted conformations for this target.

Second, quercetin was docked 100 independent times against each target to evaluate scoring reproducibility. For iNOS, the mean binding energy was −9.50 ± 0.05 kcal/mol, while for NF-κB the mean was −7.91 ± 0.22 kcal/mol. The low standard deviations indicate high scoring reproducibility for both targets, with the single-run values reported in Table 4 (−9.4 and −7.8 kcal/mol, respectively) falling within the 100-run distributions.

Molecular docking simulations were performed to evaluate the binding affinity of ENCNs and ENCs against iNOS and NF-κB. Quercetin was included as a reference compound due to its well-documented inhibitory activity against both targets [25]. The binding energies for all compounds are summarized in Table 4.

Docking results against iNOS revealed that ENCNs generally exhibited more favorable binding energies compared to erinacines. Among ENCNs, ENCN E showed the lowest binding energy (−11.5 kcal/mol), followed by ENCN A (−11.3 kcal/mol), ENCN F (−11.2 kcal/mol), ENCN Q (−10.3 kcal/mol), and ENCN L (−10.0 kcal/mol). For ENCs, ENC H displayed the most favorable binding energy (−10.0 kcal/mol), followed by ENC G (−9.7 kcal/mol), ENC S (−9.4 kcal/mol), ENC E (−9.2 kcal/mol), and ENC C (−9.1 kcal/mol).

Regarding NF-κB, overall lower binding affinities were observed compared to iNOS, consistent with the different binding site characteristics of each target. ENC D exhibited the most favorable binding energy (−8.5 kcal/mol), followed by ENC A (−7.7 kcal/mol) and ENC U (−7.5 kcal/mol). Among erinacines, ENC S showed the lowest binding energy (−7.8 kcal/mol), followed by Erinacine J (−7.5 kcal/mol) and Erinacines F, G, and H (all −7.4 kcal/mol).

Notably, certain compounds demonstrated favorable binding to both targets, suggesting potential dual-target activity. ENCN A and Q, along with ENC S, H, and G, exhibited competitive binding energies against both iNOS and NF-κB, making them promising candidates for further investigation. Based on these results, compounds with the most favorable binding energies were selected for molecular dynamics simulations to evaluate the stability of the protein–ligand complexes.

### 2.3. Molecular Docking Screening

Docking scores for the complete panel of ENCNs and ENCs against iNOS (PDB: 3E7G) and NF-κB (PDB: 8TKL) are compiled in Table 4. For both targets, the most favorable candidates within each compound class were highlighted using a rank-based cutoff (top five binding energies per target and class), providing a standardized criterion for prioritization.

For iNOS, ENCNs displayed a broader and overall more favorable affinity profile than ENCs, including multiple ligands with binding energies below −11.0 kcal/mol (Table 4). The highest-ranked ENCNs were ENCN E, A, F, Q, and L, whereas the top-scoring ENCs were ENC H, G, S, E, and C. In contrast, docking against NF-κB yielded less favorable energies and a narrower dispersion, consistent with reduced energetic separation among candidates under the same scoring framework. The highest-ranked ENCNs for NF-κB were D, A, U, I, and Q, while the top-ranked ENCs were S and J, followed by a tie at −7.4 kcal/mol (F, G, and H), all of which are indicated in Table 4.

Across both targets, a subset of compounds maintained competitive scores in iNOS and NF-κB, supporting their prioritization as potentially dual-relevant candidates within the limitations of docking-based ranking. Notably, ENCNs A and Q and ENCs S, H, and G consistently appeared among the best-scoring ligands across the two receptors (Table 4), and were therefore carried forward to the subsequent stages of the study.

### 2.4. Protein–Ligand Interaction Analysis

**Figure 2:**
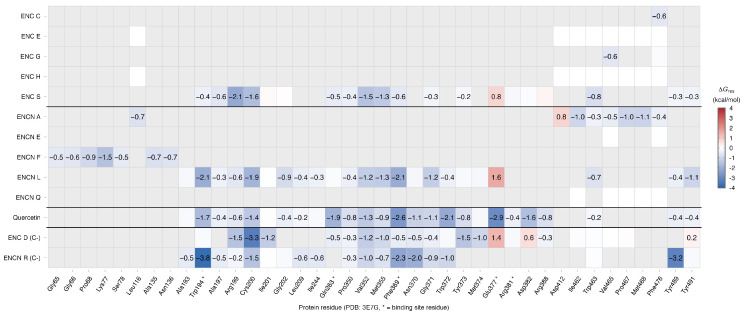
Per-residue energy decomposition heatmap for iNOS complexes. Values represent the total $\Delta G_{res}$ contribution (kcal/mol) from MM-PBSA decomposition over the 10–100 ns equilibrated trajectory. Blue indicates favorable (stabilizing) contributions; red indicates unfavorable contributions. Only residues with $|\Delta G_{res}|\;\geq \;0.5$ kcal/mol in at least one complex are displayed. Asterisks denote residues within the catalytic binding site. Top-ranked compounds and negative controls (C−) are separated by horizontal lines.

**Figure 3:**
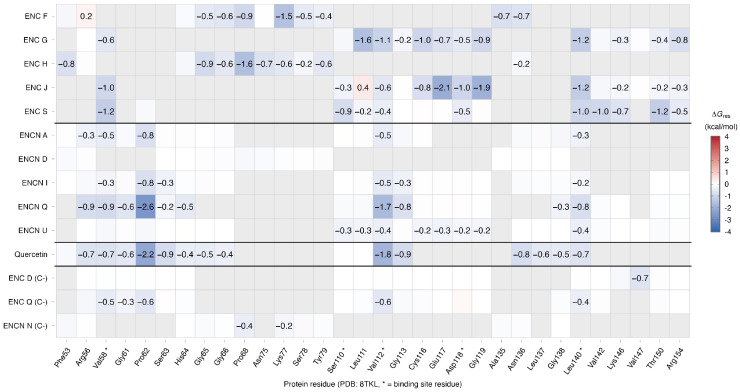
Per-residue energy decomposition heatmap for NF-κB complexes. Values represent the total $\Delta G_{res}$ contribution (kcal/mol) from MM-PBSA decomposition over the 10–100 ns equilibrated trajectory. Blue indicates favorable (stabilizing) contributions; red indicates unfavorable contributions. Only residues with $|\Delta G_{res}|\;\geq \;0.5$ kcal/mol in at least one complex are displayed. Asterisks denote residues within the binding interface. Quercetin (reference) and negative controls (C−) are separated by horizontal lines.

To complement the binding energy analysis, protein–ligand interactions were characterized for the top-ranked compounds and the reference compound quercetin against both targets. The interacting residues identified from the docked complexes are described below for each target, while the quantitative per-residue energy contributions derived from MM-PBSA decomposition over the MD trajectories are presented as heatmaps in Figure 2 and Figure 3.

For iNOS, the reference compound quercetin interacted with Trp194, Gly202, Leu209, Phe369, Trp372, and Tyr489, establishing a baseline interaction profile for comparison. Among ENCNs, Glu377 emerged as the most frequently contacted residue, being involved in interactions with four of the five top-ranked compounds (ENCNs E, F, L, and Q). Notably, Trp194 and Phe369—residues also contacted by quercetin—were recurrently observed in complexes with ENCNs A, L, and Q, suggesting a shared binding mode with the reference compound. ENCN A additionally contacted Leu209, another quercetin-interacting residue. Importantly, ENCN F interacted with Arg381, a residue previously identified as critical for iNOS inhibition [25].

Regarding erinacines, the interaction profile was characterized by consistent contacts with Trp194, Cys200, and Phe369, observed in Erinacines C, E, and H. These compounds shared two key residues (Trp194 and Phe369) with quercetin, indicating similar anchoring points within the binding cavity. Erinacine H also interacted with Gly202 and displayed contacts in the vicinity of Tyr489, mirroring the quercetin interaction pattern. Trp463 was a recurrent interacting residue among erinacines, present in complexes with Erinacines E, G, and S. Notably, Gln263, a key residue for iNOS activity [25], was identified in complexes with Erinacines C and S.

For NF-κB, quercetin established interactions with Val58, Tyr57, Pro62, Gly61, Ser63, His64, Val112, Gly113, and Leu140. This interaction profile served as a reference for evaluating the binding characteristics of ENCNs and ENCs.

Among ENCNs, Val112 was the most frequently observed residue, interacting with ENCNs A, I, and Q—matching one of the key quercetin contacts. Leu140, another quercetin-interacting residue, was present in complexes with ENCNs A and Q. ENCN Q exhibited the highest degree of overlap with quercetin, sharing four interacting residues (Val58, Val112, Leu140, and proximity to Ser110/Ser63). ENCNs A and I also contacted Pro62 and His64, respectively, further supporting a quercetin-like binding mode. ENCN D displayed a distinct interaction pattern, contacting Arg54, Gly66, and Asn136, suggesting an alternative binding site.

The erinacines exhibited a highly conserved interaction profile with NF-κB. Remarkably, Val58, Ser110, Asp118, and Leu140 were consistently observed in complexes with Erinacines G, J, and S, with Val58 and Leu140 being shared with quercetin. Erinacines F and H showed an alternative binding pattern, interacting with residues including His64 and Pro62 (in the case of Erinacine H vicinity), which partially overlap with the quercetin interaction profile.

The interaction analysis revealed that certain compounds share common binding residues with quercetin across both targets. ENCNs A and Q, which exhibited favorable binding energies against both iNOS and NF-κB (Table 4), displayed substantial overlap with the quercetin interaction profile: sharing Trp194, Phe369, Val112, and Leu140. Similarly, ENC H showed the highest similarity to quercetin for iNOS, sharing five interacting residues (Trp194, Gly202, Phe369, Trp372, Tyr489), while ENCs G, J, and S demonstrated quercetin-like interactions with NF-κB through Val58 and Leu140 contacts. These findings support the potential of these compounds as dual-target ligands for neuroinflammatory pathways, with binding properties comparable to those of the established reference compound.

The per-residue energy decomposition analysis (Figure 2 and Figure 3) provided a quantitative complement to the docking-based contact profiles described above. For iNOS, quercetin displayed the most extensive energy-decomposition profile with 15 residues contributing ≤−0.5 kcal/mol, anchored by strong interactions at Glu377 (−2.95 kcal/mol; electrostatic-driven), Phe369 (−2.58 kcal/mol; van der Waals-driven), and Trp372 (−2.15 kcal/mol). Quercetin was the only compound to establish favorable contributions at both Glu377 and Gln263 (−1.89 kcal/mol), two key catalytic residues [25]; however, given its elevated ligand RMSD (1.870 nm; Table 5), these values predominantly reflect the initial bound phase prior to ligand displacement rather than sustained pocket occupancy. Among the test compounds, ENC S (7 strong residues) and ENCN L (10 strong residues) displayed the richest profiles, sharing five residues with quercetin (Arg199, Cys200, Val352, Met355, and Phe369) that define a conserved recognition sub-site within the oxygenase domain. Notably, both showed unfavorable contributions at Glu377 (ENC S: +0.78; ENCN L: +1.61 kcal/mol), indicating a binding mode that does not engage the same charge-complementarity exploited by quercetin at this position. For NF-κB, quercetin exhibited 11 strong contacts—predominantly van der Waals-driven—anchored by Pro62 (−2.20 kcal/mol) and Val112 (−1.85 kcal/mol). ENCN Q closely replicated this profile, sharing 7 of the 11 reference residues and exceeding quercetin at Pro62 (−2.64 kcal/mol). In contrast, stable erinacine complexes (ENCs G, J, and S) engaged a distinct sub-region (Cys116–Glu117–Asp118–Gly119) with only partial overlap with the quercetin contact map, consistent with an alternative recognition surface on the p50 homodimer.

**Figure 4:**
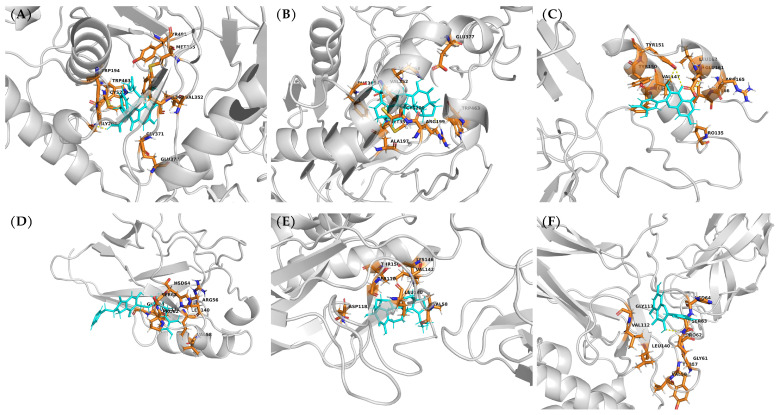
Three-dimensional visualization of representative protein–ligand complexes extracted from MD trajectories. Top row: iNOS—(**A**) ENCN L, (**B**) ENC S, (**C**) quercetin. Bottom row: NF-κB—(**D**) ENCN Q, (**E**) ENC S, (**F**) quercetin. Ligands are shown as cyan sticks; key interacting residues identified by per-residue energy decomposition are displayed as orange sticks and labeled. Yellow dashed lines indicate hydrogen bonds. The protein backbone is rendered as a gray cartoon. Molecular graphics were generated using PyMOL.

To illustrate the spatial arrangement of these interactions, representative MD snapshots of the top-performing complexes were visualized in three dimensions (Figure 4). For iNOS (Figure 4A–C), ENCN L occupies the enclosed active-site gorge in close contact with the conserved sub-site residues Cys200, Val352, Phe369, and Met355, with Glu377 positioned at the periphery of the binding cavity—consistent with the unfavorable electrostatic contribution observed in the decomposition analysis. ENC S adopts a similar orientation within the same gorge region, engaging Arg199, Cys200, and Phe369, while Trp463 provides additional van der Waals stabilization at the cavity entrance. Quercetin, despite its smaller molecular size, penetrates deeper into the cavity, establishing direct contacts with both Glu377 and Trp372 through its hydroxyl-rich scaffold, which explains its broader per-residue interaction footprint (Figure 4C). For NF-κB (Figure 4D–F), quercetin binds at the solvent-exposed surface groove anchored by Pro62, Ser63, Val112, and Leu140, with a hydrogen bond network visible between the catechol moiety and surrounding polar residues. ENCN Q occupies a closely overlapping region, consistent with its high degree of residue sharing (7 of 11) with the quercetin profile. ENC S, in contrast, sits in an adjacent pocket engaging Asp118, Ser110, Leu140, and Val58, visually confirming the alternative recognition sub-region identified by the decomposition heatmaps. Together, these three-dimensional representations corroborate the quantitative decomposition data and illustrate the distinct binding geometries adopted by each compound class within each target.

### 2.5. Molecular Dynamics Simulations

To validate the molecular docking predictions and evaluate the dynamic stability of protein–ligand complexes, 100 ns MD simulations were conducted for the top five compounds from each chemical class (ENCs and ENCNs) with both target proteins. Quercetin was included as a reference compound given its well-documented iNOS inhibitory activity. The root mean square deviation (RMSD) of ligand atomic positions relative to the initial docked conformation was monitored throughout each trajectory, with post-equilibration values (after 10 ns) used for quantitative comparisons. In addition, protein backbone RMSD, ligand–protein minimum distance, and Cα root-mean-square fluctuation (RMSF) were computed to assess protein structural integrity, confirm ligand retention within the binding site, and identify regions of enhanced flexibility, respectively. Table 5 summarizes the ligand RMSD, backbone RMSD, and minimum distance statistics for all simulated complexes, including negative controls.

**Figure 5:**
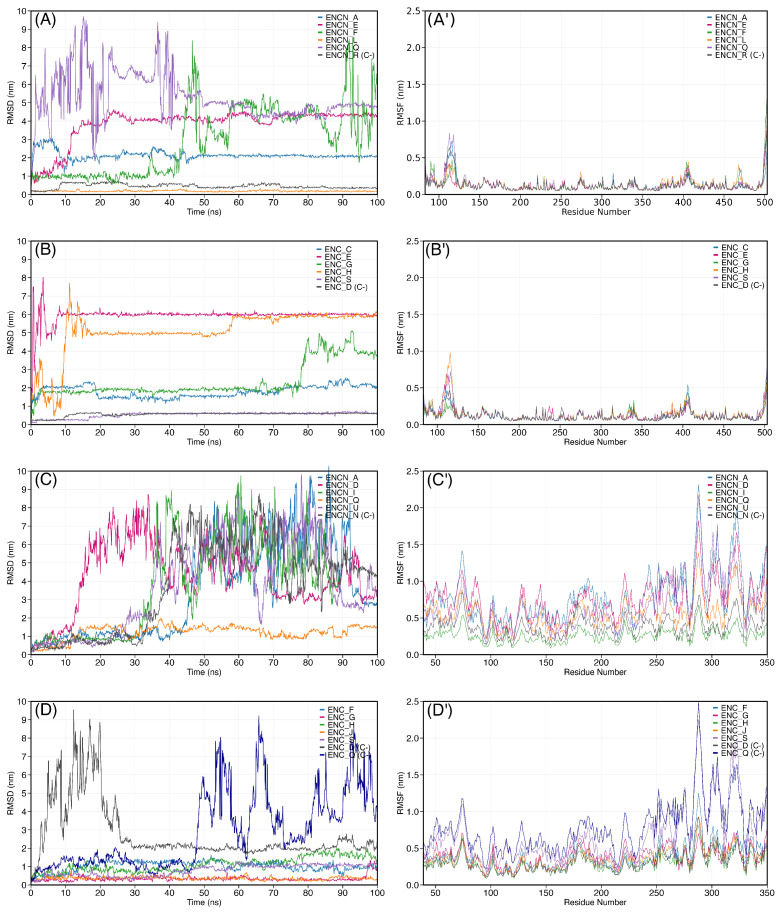
Ligand RMSD (left column) and RMSF (right column) from 100 ns MD simulations. (**A**,**A′**) ENCN–iNOS complexes; (**B**,**B′**) ENC–iNOS complexes; (**C**,**C′**) ENCN–NF-κB complexes; (**D**,**D′**) ENC–NF-κB complexes. RMSD traces are shown relative to the energy-minimized starting structure; RMSF profiles identify regions of enhanced backbone flexibility across the protein sequence.

The RMSD trajectories revealed distinct stability patterns that varied considerably depending on both the compound class and the target protein (Figure 5). For iNOS complexes, ENCN L exhibited the lowest RMSD values among all tested compounds (0.185 ± 0.035 nm), maintaining exceptional positional stability throughout the entire 100 ns simulation. This value was aproximately 10-fold lower than that observed for the reference compound quercetin (1.870 ± 0.171 nm). ENC S also demonstrated remarkable stability with iNOS, showing RMSD values of 0.573 ± 0.111 nm, which represents a 3-fold improvement compared to quercetin. ENC C displayed moderate stability with values (1.790 ± 0.315 nm) comparable to the reference compound.

The remaining ENCNs tested with iNOS showed variable behavior. ENCN A maintained relatively stable binding (2.096 ± 0.134 nm) with low fluctuation, while ENCN F exhibited an interesting trajectory pattern characterized by stable binding during the first 80 ns followed by a marked RMSD increase, suggesting a potential late-stage unbinding event. ENCNs E and Q displayed high RMSD values (4.112 and 5.334 nm, respectively), indicating poor retention within the iNOS binding site. Among ENCs, compounds H and E showed the highest instability with iNOS, with RMSD values exceeding 5 nm throughout most of the simulation.

The NF-κB simulations revealed a striking class-dependent stability pattern. All five tested ENCs formed stable complexes with RMSD values below 1.2 nm, whereas four of the five tested ENCNs exhibited high instability with values exceeding 3.5 nm (Figure 5C,D). ENCs G and J demonstrated the highest stability with NF-κB (0.345 ± 0.152 nm and 0.359 ± 0.087 nm, respectively), surpasing even the reference compound quercetin (0.753 ± 0.176 nm). ENC S maintained favorable stability (0.823 ± 0.286 nm), while ENCs F and H showed slightly higher but still acceptable RMSD values (1.078 and 1.165 nm, respectively). Among ENCNs, only ENCN Q formed a stable complex with NF-κB (1.313 ± 0.267 nm), representing the sole member of this class to maintain consistent binding throughout the simulation. The remaining ENCNs (A, U, I, and D) displayed pronounced instability characterized by RMSD values ranging from 3.8 to 4.7 nm and high standard deviations (1.5–2.6 nm), indicating significant ligand displacement and conformational fluctuations within the binding site.

**Figure 6:**
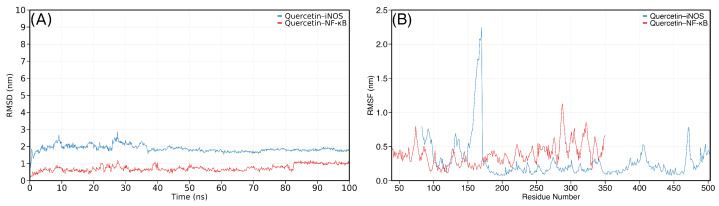
Ligand RMSD (**A**) and RMSF (**B**) for the reference compound quercetin during 100 ns MD simulations with iNOS (red) and NF-κB (blue). Quercetin displayed moderate ligand RMSD stability with both targets, with higher values for the iNOS complex (∼1.9 nm) compared to NF-κB (∼0.75 nm). The RMSF profile of the quercetin–iNOS complex showed a pronounced flexibility peak near residues 160–180, whereas the NF-κB complex exhibited more distributed flexibility across the homodimer.

The reference compound quercetin exhibited moderate stability with both targets. For iNOS, quercetin showed RMSD values of 1.870 ± 0.171 nm, positioning it in the middle range among all tested compounds. With NF-κB, quercetin demonstrated better stability (0.753 ± 0.176 nm), though it was outperformed by several erinacines. The RMSD trajectory of quercetin with both proteins remained relatively constant after initial equilibration, without major unbinding events (Figure 6).

To distinguish ligand repositioning from true dissociation, the minimum distance between the ligand and the nearest protein atom was monitored throughout each trajectory (Table 5). For iNOS, the minimum distance analysis revealed that the majority of compounds with elevated ligand RMSD values remained in contact with the protein surface (minimum distance < 0.25 nm), indicating relocation to alternative surface sites rather than complete unbinding. This was the case for ENCs C, E, G, and H, as well as ENCNs A and E, all of which maintained close protein contact despite ligand RMSD values ranging from 1.8 to 6.0 nm. Only ENCNs F and Q exhibited minimum distances exceeding 0.5 nm with high standard deviations (0.551 ± 0.734 and 0.582 ± 0.819 nm, respectively), consistent with transient dissociation events. The reference compound quercetin maintained a minimum distance of 0.219 ± 0.032 nm with iNOS, confirming persistent protein contact despite its elevated ligand RMSD. For NF-κB, the minimum distance analysis cleanly discriminated retained from dissociated complexes: all ENCs and ENCN Q maintained distances below 0.21 nm, whereas ENCNs A, D, I, and U showed mean minimum distances exceeding 1.0 nm with standard deviations above 1.0 nm, confirming complete and repeated ligand dissociation during the simulations.

**Figure 7:**
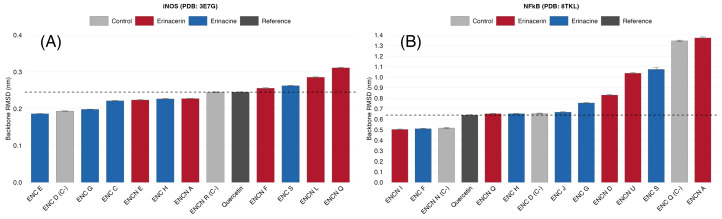
Protein backbone RMSD (post-equilibration, 10–100 ns) for all simulated complexes. (**A**) iNOS complexes; (**B**) NF-κB complexes. The dashed line indicates the quercetin reference value. Error bars represent standard deviation. Color coding: blue = erinacines, red = erinacerins, gray = negative controls, dark gray = quercetin reference.

Protein backbone RMSD analysis (Table 5; Figure 7) revealed target-dependent structural behavior. For iNOS, all complexes exhibited backbone RMSD values between 0.186 and 0.311 nm, indicating that the protein fold was well preserved throughout the simulations regardless of ligand behavior. The only exception was quercetin-iNOS, which displayed a notably higher backbone RMSD (0.564 ± 0.090 nm), suggesting that ligand repositioning in this complex was accompanied by local structural perturbation. For NF-κB, the backbone displayed inherently higher flexibility, consistent with the dimeric architecture of the p50 homodimer. Using quercetin as a reference (0.640 ± 0.134 nm), complexes with backbone RMSD values at or below this threshold included ENCNs I and Q, as well as ENCs F, H, and J. Complexes with substantially elevated backbone RMSD (ENCN A: 1.374 nm; ENC S: 1.075 nm) suggest that certain ligands may induce conformational perturbation in the NF-κB homodimer.

To evaluate dual-target potential, compounds that were simulated with both proteins were compared using a combined stability score calculated as the sum of post-equilibration RMSD values for each target (Table 6). Among the five compounds with dual-target data, ENC S exhibited the most favorable combined profile with a total score of 1.396 nm, representing a 47% improvement over quercetin (2.624 nm). ENC G showed comparable overall stability to quercetin (2.778 nm vs. 2.624 nm), though with an asymmetric distribution favoring NF-κB binding. The remaining compounds (ENCN A, ENC H, and ENCN Q) displayed combined scores exceeding 5.8 nm, primarily due to instability with one of the two targets.

The RMSF profiles (Figure 5, right column; Figure 6B) provided complementary information on protein flexibility. For iNOS, all complexes displayed a conserved flexibility pattern with peaks around residues 100–120 and the C-terminal region (>490), corresponding to exposed loop regions. The core catalytic domain (residues 200–400) remained rigid across all simulations, indicating that ligand binding or dissociation did not perturb the overall protein fold. For NF-κB, the RMSF profiles showed higher baseline fluctuations (0.3–0.5 nm) consistent with the inherent flexibility of the dimeric structure, with pronounced peaks in the C-terminal region (residues 280–340). Notably, the ENC S–NF-κB complex exhibited the highest RMSF amplitudes among the stable erinacine complexes, particularly in the C-terminal domain, which correlates with its elevated backbone RMSD (1.075 nm) and may reflect ligand-induced conformational rearrangement.

### 2.6. Binding Free Energy Calculations

To complement the molecular docking predictions and MD stability analysis, binding free energies were calculated using the MM-PBSA method for all simulated protein–ligand complexes. This approach provides a more rigorous thermodynamic assessment of binding affinity by accounting for solvation effects and entropic contributions. Table 7 summarizes the calculated binding free energies ($\Delta G_{bind}$) for both target proteins, reported as mean ± standard error of the mean (SEM) across 1000 frames extracted from the equilibrated trajectory (10–100 ns).

For iNOS, the MM-PBSA calculations revealed that ENC S exhibited the most favorable binding free energy (−24.31 kcal/mol), followed by ENCN L (−21.90 kcal/mol). Both compounds demonstrated substantially stronger binding affinities than the reference compound quercetin (−11.01 kcal/mol), with improvements of 13.30 and 10.89 kcal/mol, respectively. ENC G also showed notable affinity (−14.61 kcal/mol), approximately 3.6 kcal/mol more favorable than quercetin. Among the remaining compounds, ENCNs E and A displayed binding energies comparable to or slightly better than quercetin, while ENCs C and H, along with ENCNs Q and F, showed weaker binding affinities ranging from −7.66 to −9.83 kcal/mol.

The binding free energy results for NF-κB showed a different pattern. ENCN Q emerged as the strongest binder with $\Delta G_{bind}$ of −24.07 kcal/mol, surpassing the reference compound quercetin (−20.52 kcal/mol) by 3.55 kcal/mol. ENC G exhibited comparable affinity to quercetin (−20.38 vs. −20.52 kcal/mol), while ENCs J and H demonstrated moderately strong binding (−18.53 and −15.43 kcal/mol, respectively). Notably, the remaining ENCNs (A, U, I, and D) displayed substantially weaker binding affinities ranging from −2.32 to −7.35 kcal/mol, which correlates with the high RMSD values and complex instability observed during MD simulations for these compounds.

The MM-PBSA results showed high correlation with the MD stability data for most compounds. For iNOS, ENCN L, which exhibited the lowest RMSD values during simulation (0.185 nm), also demonstrated the second-strongest binding affinity. Similarly, ENC S showed both excellent complex stability (RMSD = 0.573 nm) and the highest binding affinity. For NF-κB, the ENCs that maintained stable complexes during MD (G, J, S, H, F) all exhibited favorable binding energies between −13.52 and −20.38 kcal/mol, while the unstable ENCNs (A, U, I, D) showed correspondingly weak binding affinities.

From a dual-target perspective, ENC S demonstrated strong binding to iNOS (−24.31 kcal/mol) with moderate affinity for NF-κB (−14.24 kcal/mol). ENC G showed a more balanced profile with good binding to both targets (−14.61 and −20.38 kcal/mol for iNOS and NF-κB, respectively). Among compounds evaluated with both targets, ENC H exhibited consistent moderate binding (−9.71 and −15.43 kcal/mol), while ENCN Q showed an interesting asymmetric profile with weak iNOS binding (−7.81 kcal/mol) but the strongest NF-κB affinity (−24.07 kcal/mol) among all tested compounds.

To assess whether the docking-based selection effectively discriminates favorable from unfavorable binders, five compounds with the lowest docking scores were subjected to the full MD/MM-PBSA pipeline as negative controls (Table 7). For NF-κB, the three negative controls (ENCs D and Q; ENCN N) yielded binding free energies between −5.28 and −9.79 kcal/mol, substantially weaker than all top-ranked ENCs (−13.52 to −20.38 kcal/mol) and the quercetin reference (−20.52 kcal/mol), confirming the discriminative capacity of the docking ranking for this target. For iNOS, however, the two negative controls yielded unexpectedly favorable binding free energies: ENCN R (−34.24 ± 0.13 kcal/mol) and ENC D (−24.67 ± 0.19 kcal/mol), both exceeding the top-ranked candidate ENC S (−24.31 kcal/mol). These compounds also maintained close protein contact throughout the simulations (minimum distance < 0.19 nm; Table 5). This result indicates that for iNOS, the docking scoring function did not reliably predict MM-PBSA binding free energies, and that the blind docking protocol—which explores the entire protein surface—may place low-scoring ligands at sites that become energetically favorable during MD relaxation. This observation underscores a recognized limitation of docking-based ranking and supports the use of MD/MM-PBSA as an essential refinement step in computational screening workflows.

## 3. Discussion

This work provides a comparative structure–activity interpretation of cyathane diterpenoids (ENCs) and isoindolinone derivatives (ENCNs) from *Hericium erinaceus* in the context of two mechanistically linked neuroinflammatory regulators, iNOS and NF-κB, which are also broadly implicated in systemic inflammation, immune regulation, cardiovascular dysfunction, and cancer progression [11]. By integrating docking-based screening with 100 ns MD simulations, per-residue energy decomposition, and MM-PBSA estimates, we identified a clear scaffold-dependent behavior across targets and prioritized ENC S and ENCN L as the most robust candidates relative to quercetin.

The chemical investigation of ENCNs was initiated by Wang et al. [16], who isolated ENCN C–L and characterized their distinctive isoindolinone scaffold. Since this foundational work, the repertoire of bioactive metabolites from *H. erinaceus* has grown substantially [33,34,35], though systematic computational studies targeting neuroinflammatory proteins remain limited. Wei et al. [25] conducted the only previous study combining molecular docking with dynamics simulations to evaluate cyathane diterpenoids from this mushroom against iNOS, using quercetin as a reference compound. Our work extends this approach by simultaneously screening both compound families against two targets that operate in sequence: NF-κB nuclear translocation triggers iNOS transcription, leading to NO overproduction and subsequent oxidative damage to neurons [5,9].

The computational analysis revealed intriguing scaffold-dependent binding patterns. ENCN L exhibited exceptional stability within the iNOS active site (RMSD = 0.185 nm), roughly ten times better than quercetin (1.87 nm), while ENC S showed both excellent stability (0.573 nm) and the strongest binding affinity (−24.31 vs. −11.01 kcal/mol for quercetin). Conversely, for NF-κB, all tested ENCs formed stable complexes (RMSD < 1.2 nm), whereas most ENCNs displayed marked instability (RMSD > 3.5 nm), with ENCN Q being the sole exception. This divergent behavior suggests that the cyathane diterpenoid scaffold common to ENCs possesses structural features complementary to the NF-κB binding pocket, while the isoindolinone core of ENCNs appears better accommodated by iNOS. Importantly, the minimum distance analysis demonstrated that elevated ligand RMSD values do not necessarily indicate dissociation from the protein. For iNOS, the majority of compounds with RMSD > 1.8 nm maintained minimum distances below 0.25 nm, indicating relocation to alternative surface sites rather than complete unbinding. Only ENCNs F and Q showed evidence of true dissociation. For NF-κB, however, the minimum distance analysis cleanly separated retained complexes (all ENCs and ENCN Q; <0.21 nm) from dissociated ones (ENCNs A, D, I, U; >1.0 nm). Protein backbone RMSD confirmed that the iNOS fold remained structurally intact across all simulations (0.186–0.311 nm), with the notable exception of the quercetin–iNOS complex (0.564 nm), while NF-κB exhibited inherently higher backbone flexibility consistent with its dimeric architecture.

The per-residue energy decomposition provided further insight into the structural determinants governing target selectivity. For iNOS, a conserved recognition sub-site comprising Arg199, Cys200, Val352, Met355, and Phe369 was shared among quercetin, ENC S, and ENCN L, suggesting that scaffold identity alone does not fully determine binding—rather, the spatial arrangement of hydrogen-bond donors/acceptors and hydrophobic groups around these core scaffolds modulates the depth and strength of pocket engagement. This is consistent with the observation that quercetin, despite its substantially lower molecular weight (302 g/mol vs. >430 g/mol for all tested ENCs and ENCNs), achieved the most extensive per-residue interaction profile with iNOS (15 strong residues), exploiting its five hydroxyl groups to establish both electrostatic contacts (Glu377, Gln263) and van der Waals stacking (Phe369, Trp372) within the relatively enclosed active-site gorge. In contrast, the larger terpenoid and isoindolinone frameworks appear to rely predominantly on van der Waals complementarity, as reflected by the unfavorable electrostatic contributions at Glu377 observed for both ENC S and ENCN L. For NF-κB, the binding interface is a relatively shallow and solvent-exposed surface groove on the p50 homodimer, which may explain why molecular size and scaffold flexibility become more relevant: the compact quercetin achieves good coverage of this surface (Pro62, Val112 as anchors), while the larger ENCs engage an adjacent sub-region (Cys116–Asp118–Gly119) that is inaccessible to quercetin, effectively extending the interaction footprint through their broader molecular envelope.

Our computational predictions align well with published experimental evidence. Lee et al. [22] showed that erinacine A inhibits iNOS expression and attenuates nitrotyrosine formation via p38 MAPK pathway inactivation in ischemic brain injury models. Later work from the same group demonstrated that erinacine A prevents LPS-induced iNOS upregulation in BV2 microglial cells while simultaneously suppressing NF-κB phosphorylation [20], directly supporting the dual-target mechanism we propose. Wang et al. [21] further showed that ENC C reduces NO production by down-regulating NF-κB and inhibiting IκBα phosphorylation. These mechanistic studies lend biological plausibility to our computational findings.

Among the compounds evaluated, ENC S stands out as the most promising dual-target candidate based on its consistently favorable performance across all computational metrics. Dual-target ligands can offer advantages over single-target agents when addressing multifactorial conditions like neuroinflammation [28]. The continued discovery of new bioactive compounds from *H. erinaceus*, including the recently reported ENCN W with neuroprotective activity [33], highlights the ongoing therapeutic interest in this natural source.

The in silico workflow demonstrated here illustrates the utility of computational methods for prioritizing natural product candidates prior to experimental validation [28,36]. Our approach successfully identified compounds with favorable predicted binding while uncovering unexpected structure-selectivity relationships. However, several methodological limitations must be acknowledged. First, the selection of candidates for MD simulations was based on a single docking-score criterion (the most favorable binding energy), which assumes that docking scoring functions can reliably rank binding affinities. This assumption is increasingly recognized as problematic: as discussed by Pantsar and Poso [37], docking was originally designed to predict binding poses rather than binding affinities, and comprehensive evaluations have demonstrated weak correlations between docking scores and experimental binding constants across diverse target–ligand systems. Our own negative control analysis provided a direct illustration of this limitation: For iNOS, two compounds excluded by the docking ranking (ENCN R and ENC D) yielded MM-PBSA binding free energies of −34.24 and −24.67 kcal/mol, respectively, exceeding the top-ranked candidate ENC S (−24.31 kcal/mol). This discrepancy may reflect the limited ability of empirical scoring functions to capture solvation effects, entropic contributions, and protein flexibility that become apparent only during MD relaxation [38]. Notably, the docking-based selection performed substantially better for NF-κB, where all three negative controls yielded weak binding energies (−5.28 to −9.79 kcal/mol) clearly separated from the top candidates, suggesting that the discriminative capacity of docking scores is target-dependent. These observations reinforce the value of the multi-tier workflow employed here, in which docking serves as an initial filter rather than a definitive ranking tool, and MD/MM-PBSA provides the necessary refinement to identify genuinely favorable complexes.

Second, only a single docked conformation per compound was used as the starting structure for MD simulations. Since the binding free energy is a collective property of all accessible conformational states [37], alternative docking poses or different initial orientations could potentially yield different MM-PBSA estimates. This limitation is particularly relevant for compounds that relocated to alternative surface sites during the simulations (as demonstrated by the minimum distance analysis), suggesting that the initial docking pose may not always correspond to the thermodynamically preferred binding mode. Third, while MM-PBSA provides a computationally efficient estimate of relative binding affinities, it does not account for conformational entropy changes upon binding, and its absolute energy values should not be interpreted as experimental binding constants [38]. The values reported here are therefore most informative for relative comparisons within the same target rather than across targets or against experimental data. Despite these limitations, the integration of negative controls, minimum distance analysis, backbone RMSD, and per-residue decomposition into the workflow enabled a substantially more nuanced interpretation of the simulation data than docking or ligand RMSD alone would provide, and the identification of these methodological caveats itself represents a constructive outcome for the design of future computational screening campaigns targeting iNOS and NF-κB.

It should be noted, more broadly, that these computational predictions require confirmation through biochemical assays and cell-based studies. Factors such as membrane permeability, metabolic stability, and blood-brain barrier penetration will ultimately determine the translational potential of these compounds as neuroinflammatory therapeutics.

## 4. Materials and Methods

### 4.1. Ligand Retrieval and Preparation

The three-dimensional structures of ENCs and ENCNs were retrieved from the PubChem database (https://pubchem.ncbi.nlm.nih.gov/, accessed on 27 January 2026) in SDF format. A total of 21 ENCNs and 18 ENCs were initially included in this study for molecular docking screening (Appendix A, Table A1). The molecular structures were imported into Avogadro software (version 1.2.0), where geometry optimization was performed using the Universal Force Field (UFF) with a steepest descent algorithm until convergence criteria were met. The energy-minimized structures were exported in MOL2 format for subsequent molecular docking studies.

Based on the molecular docking results against iNOS and NF-κB, the top-ranking compounds from each class were selected for molecular dynamics simulations. The selection criteria included binding affinity values and the quality of ligand–receptor interactions. Accordingly, seven ENCNs (A, D, E, F, L, Q, and U) and seven ENCs (C, E, F, G, H, J, and S) were chosen for further computational analysis. The molecular properties, 2D structures, and PubChem identifiers of the selected compounds are presented in Table 1 and Table 2 (Section 2).

### 4.2. Protein Retrieval and Preparation

The crystal structures of iNOS (PDB: 3E7G; resolution 2.20 Å; murine iNOS oxygenase domain in complex with AR-C124798) [39] and NF-κB p50 homodimer (PDB: 8TKL; resolution 3.00 Å) were retrieved from the RCSB Protein Data Bank (https://www.rcsb.org/, accessed on 27 January 2026). For iNOS, only chain A of the homodimeric structure was retained. Co-crystallized ligands, water molecules, and ions not essential for catalysis were removed. Hydrogen atoms were added and protonation states were assigned at pH 7.4 using UCSF Chimera [40]. The prepared protein structures served as receptors for both molecular docking and MD simulations, ensuring consistency between the two stages.

### 4.3. Physicochemical Properties and ADMET Predictions

The physicochemical properties, drug-likeness parameters, and ADMET (absorption, distribution, metabolism, excretion, and toxicity) profiles of ENCNs and ENCs selected for molecular dynamics simulations were predicted using SwissADME (http://www.swissadme.ch/, accessed on 27 January 2026) [41] and DataWarrior (version 5.5.0) [42]. All analyses were performed on 27 January 2026.

The following physicochemical and ADMET parameters were evaluated:

- **Physicochemical properties**: Molecular weight (MW), consensus LogP (cLogP), aqueous solubility (cLogS), number of hydrogen bond acceptors (HBA) and donors (HBD), topological polar surface area (TPSA), and number of rotatable bonds (n_rot_).
- **Drug-likeness**: Compliance with Lipinski’ s Rule of Five [43], which establishes that drug-like compounds should have MW ≤ 500 Da, cLogP ≤ 5, HBA ≤ 10, and HBD ≤ 5.
- **Pharmacokinetics**: Predicted gastrointestinal (GI) absorption, blood-brain barrier (BBB) permeability, and skin permeability (Log K_p_).
- **Toxicity**: Predicted mutagenicity, tumorigenicity, reproductive toxicity, and irritant potential were assessed using DataWarrior’ s built-in toxicity prediction algorithms.

### 4.4. Molecular Docking

Molecular docking simulations were performed using AutoDock Vina (version 1.2.0) [44] through the UCSF Chimera interface. A blind docking approach was employed, where the search space encompassed the entire receptor surface to identify potential binding sites without bias. The grid box was defined to cover the complete protein structure with adequate margins to allow ligand exploration of all possible binding cavities.

The docking parameters were set as follows: exhaustiveness = 10, num_modes = 10, and energy_range = 3 kcal/mol. For each ligand-receptor pair, ten binding poses were generated and ranked according to their predicted binding affinity scores. The pose with the most favorable (most negative) binding energy was selected for subsequent analysis; this top-ranked pose is designated as “model 1” throughout this manuscript. Quercetin was docked against both targets under identical conditions and served as the reference compound for all comparative analyses.

#### Docking Protocol Validation

To assess the reliability of the docking protocol, a redocking validation was performed. Quercetin was docked 100 independent times against each target protein under the same parameters described above. For each target, the mean binding energy and standard deviation across the 100 runs were computed. Additionally, for iNOS, the co-crystallized ligand AR-C124798 was extracted from PDB 3E7G and redocked into the binding site to verify that AutoDock Vina could reproduce the experimentally observed binding pose. The root-mean-square deviation between the redocked and co-crystallized ligand conformations was calculated using UCSF Chimera https://www.cgl.ucsf.edu/chimera/.

### 4.5. Protein–Ligand Interaction Analysis

The identification of protein–ligand interactions is essential to understanding the molecular basis of binding affinity and to validating the predicted binding modes. While docking scores provide a quantitative estimate of binding strength, the characterization of specific intermolecular contacts allows for the comparison of binding profiles among compounds and the identification of key residues involved in ligand recognition [28].

Protein–ligand interactions from the docked complexes were characterized using AutoDock Tools 1.5.7 [45]. For each protein–ligand complex, the best-scored docked pose (model 1, lowest binding energy) in PDBQT format was loaded, and a distance-based contact analysis was performed using the default cutoff of 4.0 Å between any ligand atom and any protein residue atom. Residues with at least one atomic pair below this threshold were classified as interacting. The interacting residues were manually recorded for all ENCNs and ENCs with both iNOS and NF-κB. Particular attention was given to interactions with residues previously reported as critical for iNOS inhibition, including Gln263, Glu377, and Arg381 [25].

### 4.6. Molecular Dynamics Simulations

Molecular dynamics (MD) simulations were performed to assess the dynamic stability of the top-ranked ligand–receptor complexes identified from molecular docking. All simulations were conducted using GROMACS (versions 2024.4) [46] with GPU acceleration (NVIDIA RTX 3080). The CHARMM36m force field [47] was employed for protein parameterization, while ligand topology and parameters were generated using the CHARMM General Force Field (CGenFF) through the CGenFF server (https://cgenff.com/, accessed on 27 January 2026).

Each protein–ligand complex was centered in a cubic simulation box with a minimum distance of 1.0 nm between the solute and box edges. The systems were solvated with TIP3P water molecules [48] and neutralized by adding Na^+^ and Cl^−^ ions to achieve a physiological ionic strength of 0.15 M. Energy minimization was subsequently performed using the steepest descent algorithm for 5000 steps or until the maximum force converged below 1000 kJ/mol/nm.

Following minimization, systems were equilibrated in two sequential stages with position restraints applied to solute heavy atoms (force constant = 1000 kJ/mol/nm^2^). First, NVT equilibration was conducted for 1 ns at 300 K using the V-rescale thermostat [49] with a coupling constant of 0.1 ps. Subsequently, position restraints were gradually released during NPT equilibration at 300 K and 1 bar using the Parrinello-Rahman barostat [50] with a coupling constant of 2.0 ps.

Production MD simulations were performed for 100 ns under NPT conditions without position restraints. The LINCS algorithm [51] was applied to constrain all bonds involving hydrogen atoms, allowing a 2 fs integration time step. Long-range electrostatic interactions were calculated using the Particle Mesh Ewald (PME) method [52] with a real-space cutoff of 1.2 nm, while van der Waals interactions were truncated at 1.2 nm with a switching function applied from 1.0 nm. Trajectory coordinates were saved every 100 ps for structural analysis, and energetic quantities were recorded every 10 ps.

#### 4.6.1. Complexes Simulated

Based on the molecular docking results, the following complexes were subjected to MD simulations:

**iNOS complexes (10 total):**

- Erinacerins: A, E, F, L, and Q
- Erinacines: C, E, G, H, and S

**NF-κB complexes (10 total):**

- Erinacerins: A, D, I, Q, and U
- Erinacines: F, G, H, J, and S

Notably, ENCNs A and Q, along with ENCs S, H, and G, were simulated with both protein targets, enabling comparative analysis of their binding behavior across the two neuroinflammation-related proteins. Quercetin was also simulated with both iNOS and NF-κB under identical conditions to serve as the reference for MD stability and binding free energy comparisons.

#### 4.6.2. Negative Control Simulations

To evaluate whether the docking-based selection effectively discriminates favorable from unfavorable binders, five compounds with the lowest (least favorable) docking scores were subjected to the full MD/MM-PBSA pipeline as negative controls. The negative control set comprised ENCNs M and N (iNOS) and ENCNs B, H, and J (NF-κB), as well as ENCs D and Q (NF-κB). These complexes were simulated and analyzed under identical conditions to those described for the top-ranked candidates.

#### 4.6.3. Trajectory Analysis

Trajectory analyses were performed using GROMACS built-in tools. Ligand RMSD was calculated using gmx rms by least-squares fitting on the protein backbone atoms (Cα, C, N) and computing the deviation on all ligand heavy atoms, relative to the energy-minimized starting structure. Protein backbone RMSD was computed separately by fitting and calculating the deviation on backbone atoms (Cα, C, N) of the entire protein chain. Root-mean-square fluctuation (RMSF) of Cα atoms was obtained using gmx rmsf to identify regions of enhanced flexibility. Ligand–protein minimum distances were calculated using gmx mindist with the -or flag, selecting the ligand group against the full protein group to obtain per-residue minimum distances while avoiding periodic boundary condition artifacts. All analyses were performed on the post-equilibration portion of the trajectories (10–100 ns), after confirming system equilibration from RMSD convergence.

### 4.7. Binding Free Energy Calculations

Binding free energies were calculated using the Molecular Mechanics Poisson–Boltzmann Surface Area (MM-PBSA) method implemented in gmx_MMPBSA (version 1.6.4) [53]. Calculations were performed on frames extracted from the equilibrated portion of each MD trajectory (10–100 ns).

The binding free energy ($\Delta G_{bind}$) was computed according to:

(1) $$\Delta G_{bind}=G_{complex}-G_{receptor}-G_{ligand}$$

where each free energy term comprises molecular mechanics energy ($E_{MM}$) and solvation free energy ($G_{solv}$) contributions. The molecular mechanics component includes electrostatic and van der Waals interactions, while the solvation free energy consists of polar and non-polar terms calculated using the Poisson-Boltzmann equation and solvent-accessible surface area methods, respectively. Default parameters were used as implemented in gmx_MMPBSA. Results are reported as mean ± standard error of the mean (SEM) across the analyzed frames.

#### Per-Residue Energy Decomposition

To quantify individual residue contributions to binding free energy, the interaction decomposition scheme implemented in gmx_MMPBSA was employed (idecomp = 1). Decomposition was performed for all residues within 12 Å of the ligand (print_res = “within 12”), capturing both direct contact and second-shell electrostatic contributions. The resulting per-residue energy values (kcal/mol) were used to construct interaction heatmaps for each target protein, enabling quantitative comparison of binding profiles across compounds.

### 4.8. Data Visualization and Statistical Analysis

Molecular graphics and structural visualizations were generated using PyMOL (version 2.5; Schrödinger, LLC) and UCSF Chimera [40]. RMSD, RMSF, and backbone RMSD trajectory plots and descriptive statistics, including mean and standard deviation for post-equilibration values (10–100 ns), were computed using R (version 4.4.2; R Core Team, 2024) [54]. Per-residue energy decomposition heatmaps were generated in R using the ggplot2 package (version 3.5.1) [55].

## 5. Conclusions

In summary, our computational results identify ENC S as the most promising dual-target candidate against iNOS and NF-κB, combining a markedly favorable predicted binding affinity for iNOS compared with quercetin (−24.31 vs. −11.01 kcal/mol) with stable interactions across both proteins throughout the simulations. In addition, ENCN L exhibited exceptional positional stability within the iNOS binding pocket, showing the lowest ligand RMSD among all tested complexes. Minimum distance analysis confirmed that the majority of iNOS complexes with elevated ligand RMSD remained in contact with the protein, while NF-κB complexes showed clear discrimination between retained and dissociated ligands. Overall, these findings support a scaffold-dependent interaction profile in which ENCs tend to maintain stable complexes with both targets, whereas ENCNs appear better accommodated by iNOS but are generally unstable within the NF-κB site. Per-residue energy decomposition further showed that this selectivity is modulated by the spatial arrangement of functional groups relative to the distinct binding site topologies of each target. These in silico insights provide a focused basis for subsequent biochemical and cell-based validation to assess the therapeutic potential of *H. erinaceus* metabolites in neuroinflammatory pathways.

Taken together, the present findings are directionally consistent with a dual-target anti-inflammatory profile proposed for selected ENCs in neuroinflammatory models. Accordingly, ENC S is prioritized for follow-up validation using enzymatic inhibition assays and cell-based readouts of NF-κB activity and NO production, while ENCN L warrants further evaluation as a selective iNOS-oriented scaffold. Notably, the negative control analysis revealed that docking scores did not reliably predict MM-PBSA binding free energies for iNOS, underscoring the need for MD-based refinement in computational screening workflows.

Overall, this in silico workflow provides a rational basis to prioritize *H. erinaceus* metabolites for targeted experimental testing and mechanistic refinement of neuroinflammatory pathways. 

## Figures and Tables

**Table 1:** Molecular properties and 2D structures of ENCNs from *Hericium erinaceus* selected for molecular dynamics simulations.

Compound	2D Structure	PubChem CID	Molecular Formula
Erinacerin A	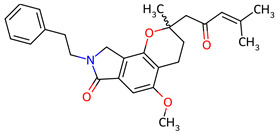	11524866	C_27_H_31_NO_4_
Erinacerin D	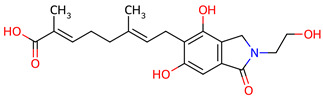	101910490	C_20_H_25_NO_6_
Erinacerin E	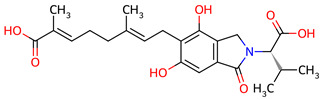	101910491	C_23_H_29_NO_7_
Erinacerin F	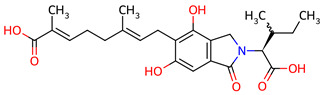	101910492	C_24_H_31_NO_7_
Erinacerin L	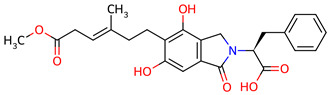	139585426	C_25_H_27_NO_7_
Erinacerin Q	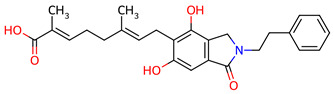	132599515	C_26_H_29_NO_5_
Erinacerin U	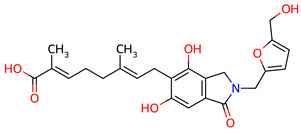	146683976	C_24_H_27_NO_7_

**Table 2:** Molecular properties and 2D structures of ENCs from *Hericium erinaceus* selected for molecular dynamics simulations.

Compound	2D Structure	PubChem CID	Molecular Formula
Erinacine C	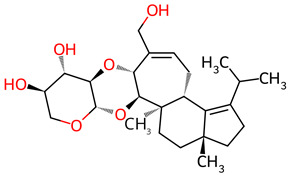	10252378	C_25_H_38_O_6_
Erinacine E	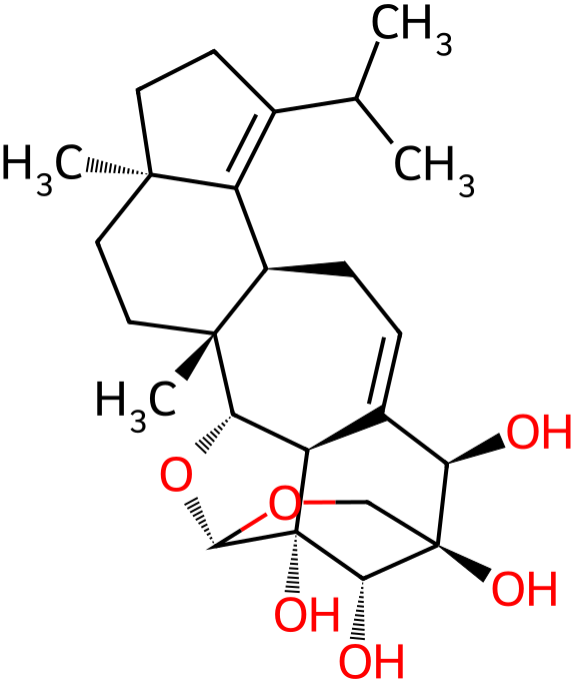	11743545	C_25_H_36_O_6_
Erinacine F	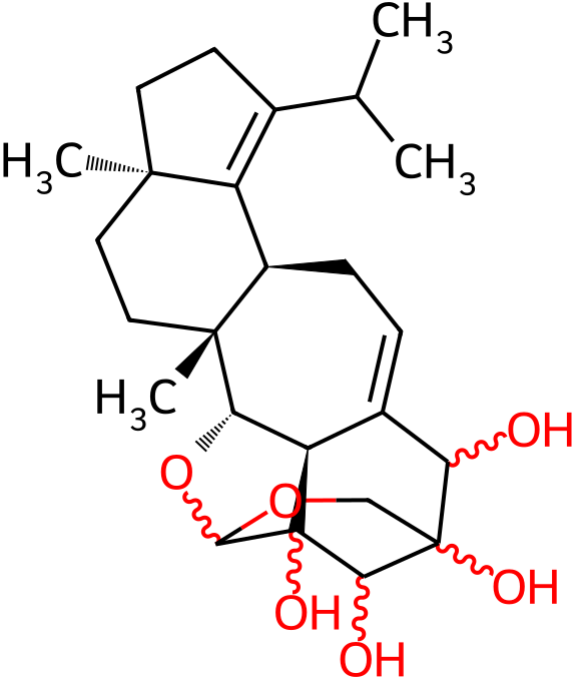	10342778	C_25_H_36_O_6_
Erinacine G	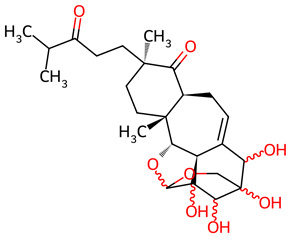	10552092	C_25_H_36_O_8_
Erinacine H ^a^	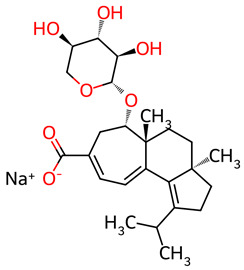	102012688	C_25_H_36_O_7_
Erinacine J	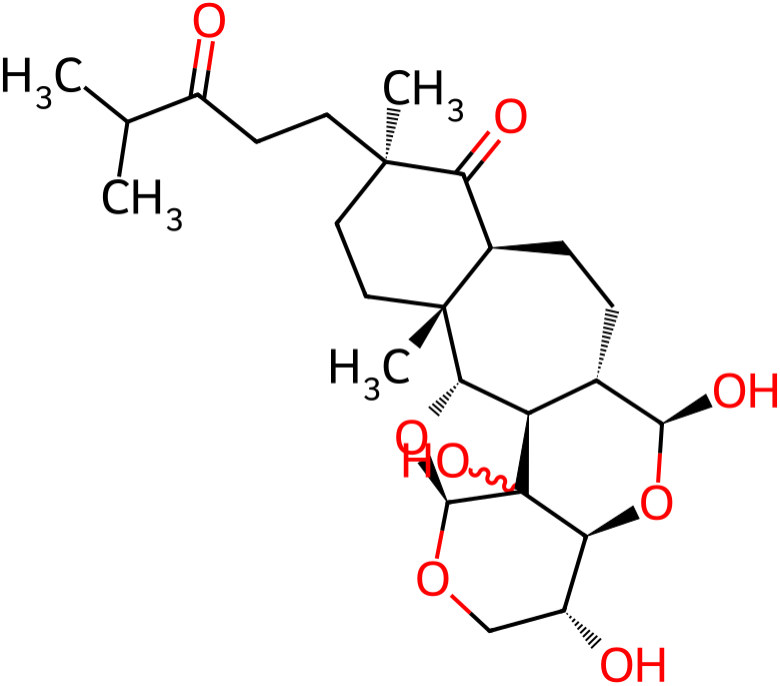	16085267	C_25_H_38_O_8_
Erinacine S	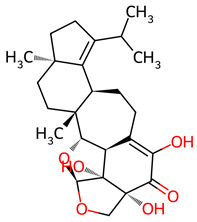	127047879	C_25_H_34_O_6_

^a^ PubChem reports Erinacine H as the sodium carboxylate salt ($C_{25}H_{35}O_{7}^{-}$, CID 102012688). The neutral carboxylic acid form is reported here for consistency; protonation states at physiological pH (7.4) were assigned during system preparation as described in Section 2.

**Table 3:** Physicochemical properties, ADME predictions, and in silico toxicity assessment of ENCNs and ENCs selected for molecular dynamics simulations.

Compound	MW	cLogP	cLogS	Solubility	GI	BBB	Ro5	DL	Irr.
	(g/mol)								
Erinacerin A	433.54	4.91	−4.70	Low	High	Yes	Yes	1.69	High
Erinacerin D	375.42	2.53	−2.09	—	—	—	—	2.04	None
Erinacerin E	431.48	3.21	−2.88	—	—	—	—	0.57	None
Erinacerin F	445.51	3.67	−3.15	—	—	—	—	3.60	None
Erinacerin I	349.38	1.69	−1.78	Soluble	High	No	Yes	−4.91	None
Erinacerin L	453.48	3.15	−3.27	Moderate soluble	High	No	Yes	−4.59	None
Erinacerin Q	435.51	4.90	−3.73	—	—	—	—	1.89	None
Erinacerin U	441.47	3.11	−3.21	—	—	—	—	2.45	None
Erinacine C	434.57	2.40	−3.59	Soluble	High	No	Yes	−9.41	None
Erinacine E	432.55	2.02	−3.35	Soluble	High	No	Yes	−5.55	High
Erinacine F	432.55	1.93	−3.35	Soluble	High	No	Yes	−5.55	High
Erinacine G	464.55	0.98	−3.09	Soluble	High	No	Yes	−5.89	High
Erinacine H	447.54	2.28	−3.22	Soluble	High	No	Yes	−10.75	None
Erinacine J	466.56	1.57	−3.62	Soluble	High	No	Yes	−6.54	High
Erinacine S	430.53	2.58	−3.73	Soluble	High	No	Yes	−5.24	None

Note: Consensus cLogP. MW: molecular weight (g/mol); cLogS: calculated aqueous solubility; GI: gastrointestinal absorption; BBB: blood–brain barrier permeability; Ro5: Lipinski’ s Rule of Five compliance; DL: drug-likeness score; Irr.: irritant. All compounds showed no mutagenic, tumorigenic, or reproductive effects. (—) indicates data not available.

**Table 4:** Molecular docking binding energies (kcal/mol) for ENCNs and ENCs against iNOS (PDB: 3E7G) and NF-κB (PDB: 8TKL).

Erinacerin	iNOS	NF-κB	Erinacine	iNOS	NF-κB
Quercetin (Ref.)	−9.4	−7.8			
A	−11.3	−7.7	A	−8.7	−6.7
B	−7.6	−5.8	B	−9.0	−7.0
C	−8.2	−6.3	C	−9.1	−7.0
D	−9.7	−8.5	D	−7.8	−6.2
E	−11.5	−6.3	E	−9.2	−6.9
F	−11.2	−6.5	F	−8.4	−7.4
G	−7.8	−6.1	G	−9.7	−7.4
H	−7.4	−5.8	H	−10.0	−7.4
I	−7.8	−6.8	I	−8.7	−6.5
J	−7.2	−5.7	J	−9.0	−7.5
K	−9.4	−6.5	K	−8.1	−6.6
L	−10.0	−6.1	P	−8.3	−6.9
M	−7.8	−5.8	Q	−8.6	−6.2
N	−7.5	−5.2	R	−8.2	−7.1
O	−7.7	−5.7	S	−9.4	−7.8
P	−9.2	−5.7	T	−8.5	−6.7
Q	−10.3	−6.6	U	−8.2	−6.4
R	−7.0	−6.2	V	−8.1	−6.8
S	−8.5	−6.2			
T	−8.4	−5.9			
U	−9.2	−7.5			

Binding energies obtained from AutoDock Vina (model 1). More negative values indicate stronger predicted binding affinity. Values indicate the top five binding energies for each target within each compound class.

**Table 5:** Summary of molecular dynamics trajectory metrics from 100 ns simulations. Ligand RMSD, protein backbone RMSD, and ligand–protein minimum distance are reported as post-equilibration means ± SD (10–100 ns). Compounds are ranked by ligand RMSD within each target.

Target	Class	ID	Ligand RMSD (nm)	BB RMSD (nm)	Min. Dist. (nm)
iNOS	Erinacerin	L	0.185 ± 0.035	0.286 ± 0.036	0.183 ± 0.014
	Erinacine	S	0.573 ± 0.111	0.263 ± 0.022	0.187 ± 0.016
	Erinacine	C	1.790 ± 0.315	0.222 ± 0.023	0.200 ± 0.018
	**Ref.**	**Qrc**	**1.870 ± 0.171**	**0.564 ± 0.090**	**0.219 ± 0.032**
	Erinacerin	A	2.096 ± 0.134	0.228 ± 0.018	0.209 ± 0.015
	Erinacine	G	2.433 ± 0.961	0.198 ± 0.017	0.212 ± 0.055
	Erinacerin	F	3.128 ± 1.831	0.256 ± 0.048	0.551 ± 0.734
	Erinacerin	E	4.112 ± 0.362	0.224 ± 0.045	0.203 ± 0.026
	Erinacerin	Q	5.334 ± 1.222	0.311 ± 0.045	0.582 ± 0.819
	Erinacine	H	5.436 ± 0.487	0.227 ± 0.043	0.261 ± 0.317
	Erinacine	E	6.001 ± 0.053	0.186 ± 0.019	0.206 ± 0.013
	Erinacine	D ^†^	0.590 ± 0.040	0.194 ± 0.018	0.185 ± 0.014
	Erinacerin	R ^†^	0.472 ± 0.109	0.245 ± 0.033	0.173 ± 0.009
NF-κB	Erinacine	G	0.345 ± 0.152	0.755 ± 0.163	0.181 ± 0.015
	Erinacine	J	0.359 ± 0.087	0.668 ± 0.122	0.176 ± 0.012
	**Ref.**	**Qrc**	**0.753 ± 0.176**	**0.640 ± 0.134**	**0.206 ± 0.020**
	Erinacine	S	0.823 ± 0.286	1.075 ± 0.450	0.192 ± 0.017
	Erinacine	F	1.078 ± 0.203	0.511 ± 0.127	0.199 ± 0.017
	Erinacine	H	1.165 ± 0.361	0.653 ± 0.122	0.200 ± 0.014
	Erinacerin	Q	1.313 ± 0.267	0.653 ± 0.144	0.204 ± 0.012
	Erinacerin	A	3.795 ± 2.556	1.374 ± 0.289	1.239 ± 1.280
	Erinacerin	U	3.996 ± 2.286	1.038 ± 0.200	1.061 ± 1.060
	Erinacerin	I	4.127 ± 2.459	0.503 ± 0.124	1.352 ± 1.218
	Erinacerin	D	4.664 ± 1.471	0.830 ± 0.201	1.163 ± 1.040
	Erinacine	D ^†^	2.671 ± 1.511	0.655 ± 0.142	0.493 ± 0.771
	Erinacine	Q ^†^	3.075 ± 2.048	1.346 ± 0.198	0.978 ± 1.059
	Erinacerin	N ^†^	4.157 ± 2.354	0.517 ± 0.164	1.305 ± 1.146

BB RMSD: backbone RMSD (Cα, C, N atoms). Min. Dist.: minimum ligand–protein distance. Qrc: Quercetin. ^†^ Negative control compounds (lowest docking scores)

**Table 6:** Dual-target stability assessment for compounds evaluated with both iNOS and NF-κB. The combined score represents the sum of post-equilibration RMSD values for both targets.

Compound	iNOS RMSD (nm)	NF-κB RMSD (nm)	Combined Score (nm)	vs. Quercetin
Erinacine S	0.573 ± 0.111	0.823 ± 0.286	1.396	−47%
**Quercetin**	**1.870 ± 0.171**	**0.753 ± 0.176**	**2.624**	**Reference**
Erinacine G	2.433 ± 0.961	0.345 ± 0.152	2.778	+6%
Erinacerin A	2.096 ± 0.134	3.795 ± 2.556	5.890	+124%
Erinacine H	5.436 ± 0.487	1.165 ± 0.361	6.600	+152%
Erinacerin Q	5.334 ± 1.222	1.313 ± 0.267	6.646	+153%

**Table 7:** Binding free energies ($\Delta G_{bind}$) calculated using MM-PBSA for iNOS and NF-κB complexes. Values represent mean ± SEM (kcal/mol). Compounds are ranked by binding affinity within each target.

iNOS (PDB: 3E7G)			NF-κB (PDB: 8TKL)		
Class	ID	$\Delta G_{\mathrm{bind}}$ (kcal/mol)	Class	ID	$\Delta G_{\mathrm{bind}}$ (kcal/mol)
Erinacine	S	−24.31 ± 0.16	Erinacerin	Q	−24.07 ± 0.11
Erinacerin	L	−21.90 ± 0.10	**Reference**	**Quercetin**	**−20.52 ± 0.15**
Erinacine	G	−14.61 ± 0.22	Erinacine	G	−20.38 ± 0.16
Erinacerin	E	−12.07 ± 0.18	Erinacine	J	−18.53 ± 0.12
Erinacerin	A	−11.79 ± 0.14	Erinacine	H	−15.43 ± 0.13
**Reference**	**Quercetin**	**−11.01 ± 0.19**	Erinacine	S	−14.24 ± 0.11
Erinacine	E	−10.89 ± 0.13	Erinacine	F	−13.52 ± 0.16
Erinacine	C	−9.83 ± 0.15	Erinacerin	A	−7.35 ± 0.25
Erinacine	H	−9.71 ± 0.20	Erinacerin	U	−5.57 ± 0.25
Erinacerin	Q	−7.81 ± 0.22	Erinacerin	I	−4.31 ± 0.22
Erinacerin	F	−7.66 ± 0.16	Erinacerin	D	−2.32 ± 0.15
Erinacerin	R ^†^	−34.24 ± 0.13	Erinacine	D ^†^	−9.79 ± 0.21
Erinacine	D ^†^	−24.67 ± 0.19	Erinacine	Q ^†^	−5.99 ± 0.23
			Erinacerin	N ^†^	−5.28 ± 0.21

SEM: standard error of the mean. ^†^ Negative control compounds (lowest docking scores), included to assess the discriminative capacity of the docking-based selection.

## Data Availability

The original contributions presented in this study are included in the article. Further inquiries can be directed to the corresponding authors.

## Abbreviations

The following abbreviations are used in this manuscript:

ADME	Absorption, Distribution, Metabolism, and Excretion
ADMET	Absorption, Distribution, Metabolism, Excretion, and Toxicity
Akt	Protein Kinase B
APC	Article Processing Charge
BB	Backbone
BBB	Blood–Brain Barrier
BDNF	Brain-Derived Neurotrophic Factor
BV2	Murine Microglial Cell Line (BV2)
cLogP	Calculated Octanol/Water Partition Coefficient
cLogS	Calculated Aqueous Solubility
CM	Conditioned Medium
CTX TNA2	Murine Astrocyte Cell Line (CTX TNA2)
DLS	Dynamic Light Scattering
ENC	Erinacines
ENCN	Erinacerins
FST	Forced Swimming Test
GI	Gastrointestinal
GSK-3β	Glycogen Synthase Kinase 3 Beta
HBA	Hydrogen Bond Acceptor(s)
HBD	Hydrogen Bond Donor(s)
HO-1	Heme Oxygenase-1
IC_50_	Half-Maximal Inhibitory Concentration
IFN-γ	Interferon Gamma
IKK	IκB Kinase
IκBα	Inhibitor of NF-κB Alpha
IL-1β	Interleukin 1 Beta
IL-6	Interleukin 6
iNOS	Inducible Nitric Oxide Synthase
J774A.1	Murine Macrophage Cell Line (J774A.1)
JNK	c-Jun N-terminal Kinase
LPS	Lipopolysaccharide
MAPK	Mitogen-Activated Protein Kinase
MD	Molecular Dynamics
MM–PBSA	Molecular Mechanics/Poisson–Boltzmann Surface Area
MW	Molecular Weight
N2a	Mouse Neuroblastoma Cell Line (Neuro-2a)
NF-κB	Nuclear Factor Kappa B
NGF	Nerve Growth Factor
NO	Nitric Oxide
Nrf2	Nuclear Factor Erythroid 2-Related Factor 2
PDB	Protein Data Bank
PI3K	Phosphoinositide 3-Kinase
PK	Pharmacokinetics
RCSB	Research Collaboratory for Structural Bioinformatics
Rg	Radius of Gyration
RMSD	Root Mean Square Deviation
RMSF	Root Mean Square Fluctuation
Ro5	Lipinski’s Rule of Five
RS	Restraint Stress
SDF	Structure Data File
SEM	Standard Error of the Mean
SH-SY5Y	Human Neuroblastoma Cell Line (SH-SY5Y)
SN	Substantia Nigra
STAT1	Signal Transducer and Activator of Transcription 1
TH	Tyrosine Hydroxylase
TNF-α	Tumor Necrosis Factor Alpha
TPSA	Topological Polar Surface Area
TrkB	Tropomyosin Receptor Kinase B
UFF	Universal Force Field
Vina	AutoDock Vina

## Appendix A. Ligand Library and Structural Descriptors

For transparency and reproducibility, this Appendix compiles the complete ligand library used in the docking screening stage, including both isoindolinone derivatives (erinacerins) and cyathane diterpenoids (erinacines) from *Hericium erinaceus*. The table reports PubChem identifiers, molecular formulae, and molecular weights, serving as a traceable reference for compound retrieval and downstream processing.

All structures were obtained from PubChem in SDF format and subsequently standardized through geometry optimization in Avogadro using the Universal Force Field (UFF) under a steepest-descent scheme, consistent with the protocol described in the Section 4. This list supports the screening workflow by documenting the exact chemical entities considered prior to the selection of candidates for molecular dynamics simulations.

**Table A1:** Molecular properties of erinacerins and erinacines from *Hericium erinaceus* used in this study. All structures were retrieved from PubChem and optimized using the Universal Force Field (UFF) in Avogadro.

Compound	Class	PubChem CID	Molecular Formula	MW (g/mol)
Erinacerin A	Erinacerin	11524866	C_27_H_31_NO_4_	433.23
Erinacerin B	Erinacerin	11515478	C_19_H_24_O_5_	332.16
Erinacerin C	Erinacerin	101910489	C_18_H_21_NO_5_	331.14
Erinacerin D	Erinacerin	101910490	C_20_H_25_NO_6_	375.17
Erinacerin E	Erinacerin	101910491	C_23_H_29_NO_7_	431.19
Erinacerin F	Erinacerin	101910492	C_24_H_31_NO_7_	445.21
Erinacerin G	Erinacerin	101910493	C_16_H_19_NO_5_	305.13
Erinacerin H	Erinacerin	101910494	C_16_H_19_NO_5_	305.13
Erinacerin I	Erinacerin	101910495	C_18_H_23_NO_6_	349.15
Erinacerin J	Erinacerin	101910496	C_18_H_23_NO_6_	349.15
Erinacerin K	Erinacerin	139588050	C_25_H_27_NO_7_	453.18
Erinacerin L	Erinacerin	139585426	C_25_H_27_NO_7_	453.18
Erinacerin M	Erinacerin	132599511	C_13_H_15_NO_4_	249.10
Erinacerin N	Erinacerin	132599512	C_19_H_28_N_2_O_6_	380.19
Erinacerin O	Erinacerin	132599513	C_19_H_28_N_2_O_6_	380.19
Erinacerin P	Erinacerin	132599514	C_22_H_26_N_2_O_6_	414.18
Erinacerin Q	Erinacerin	132599515	C_26_H_29_NO_5_	435.20
Erinacerin R	Erinacerin	132599516	C_17_H_21_NO_5_	319.14
Erinacerin S	Erinacerin	132599517	C_18_H_19_NO_6_	345.12
Erinacerin T	Erinacerin	139584511	C_16_H_17_NO_6_	319.11
Erinacerin U	Erinacerin	146683976	C_24_H_27_NO_7_	441.18
Erinacine A	Erinacine	10410568	C_25_H_36_O_6_	432.25
Erinacine B	Erinacine	9824135	C_25_H_36_O_6_	432.25
Erinacine C	Erinacine	10252378	C_25_H_38_O_6_	434.27
Erinacine D	Erinacine	9912965	C_27_H_42_O_7_	478.29
Erinacine E	Erinacine	11743545	C_25_H_36_O_6_	432.25
Erinacine F	Erinacine	10342778	C_25_H_36_O_6_	432.25
Erinacine G	Erinacine	10552092	C_25_H_36_O_8_	464.24
Erinacine H	Erinacine	102012688	C_25_H_36_O_7_ ^a^	448.25
Erinacine I	Erinacine	165365826	C_22_H_32_O_4_	360.23
Erinacine J	Erinacine	16085267	C_25_H_38_O_8_	466.26
Erinacine K	Erinacine	16085268	C_27_H_42_O_8_	494.29
Erinacine P	Erinacine	15886639	C_27_H_40_O_8_	492.27
Erinacine Q	Erinacine	10206893	C_27_H_42_O_8_	494.29
Erinacine R	Erinacine	102392649	C_27_H_38_O_9_	506.25
Erinacine S	Erinacine	127047879	C_25_H_34_O_6_	430.24
Erinacine T	Erinacine	139589560	C_25_H_38_O_7_	450.26
Erinacine U	Erinacine	139589559	C_26_H_40_O_7_	464.28
Erinacine V	Erinacine	139589561	C_26_H_40_O_7_	464.28

^a^ PubChem reports erinacine H as the deprotonated species $C_{25}H_{35}O_{7}^{-}$ (MW 447.24 g/mol; CID 102012688). The neutral form C_25_H_36_O_7_ (MW 448.25 g/mol) was used throughout this study for consistency with the protonation states assigned during force field parameterization.

## References

[1] Wilson D.M., Cookson M.R., Van Den Bosch L., Bhattacharyya A., Bhattacharyya S., Bhattacharyya S., Li Y., Bhattacharyya R., Chen S. (2023). Hallmarks of neurodegenerative diseases. Cell.

[2] World Health Organization (2025). Dementia. WHO Fact Sheet. https://www.who.int/news-room/fact-sheets/detail/dementia.

[3] World Health Organization (2023). Parkinson Disease. WHO Fact Sheet. https://www.who.int/news-room/fact-sheets/detail/parkinson-disease.

[4] GBD 2019 Dementia Forecasting Collaborators (2022). Estimation of the global prevalence of dementia in 2019 and forecasted prevalence in 2050: An analysis for the Global Burden of Disease Study 2019. Lancet Public Health.

[5] DiSabato D.J., Quan N., Bhattacharyya S.K. (2016). Neuroinflammation: The devil is in the details. J. Neurochem..

[6] Muzio L., Viotti A., Bhattacharyya G. (2021). Microglia in neuroinflammation and neurodegeneration: From understanding to therapy. Front. Neurosci..

[7] Shabab T., Khanabdali R., Moghadamtousi S.Z., Kadir H.A., Mohan G. (2017). Neuroinflammation pathways: A general review. Int. J. Neurosci..

[8] Bhardwaj S., Grewal A.K., Singh S., Bhattacharyya S. (2024). An insight into the concept of neuroinflammation and neurodegeneration in Alzheimer’s disease: Targeting molecular approach Nrf2, NF-*κ*B, and CREB. Inflammopharmacology.

[9] KRÖncke K.-D., Fehsel K., Kolb-Bachofen V. (1998). Inducible nitric oxide synthase in human diseases. Clin. Exp. Immunol..

[10] Calabrese V., Mancuso C., Calvani M., Rizzarelli E., Butterfield D.A., Stella A.M.G. (2007). Nitric oxide in the central nervous system: Neuroprotection versus neurotoxicity. Nat. Rev. Neurosci..

[11] Guo Q., Jin Y., Chen X., Ye X., Shen X., Lin M., Zeng C., Zhou T., Zhang J. (2024). NF-*κ*B in biology and targeted therapy: New insights and translational implications. Signal Transduct. Target. Ther..

[12] Newman D.J., Cragg G.M. (2020). Natural products as sources of new drugs over the nearly four decades from 01/1981 to 09/2019. J. Nat. Prod..

[13] Friedman M. (2015). Chemistry, nutrition, and health-promoting properties of *Hericium erinaceus* (lion’s mane) mushroom fruiting bodies and mycelia and their bioactive compounds. J. Agric. Food Chem..

[14] Venturella G., Ferraro V., Cirlincione F., Gargano M.L. (2021). Medicinal mushrooms: Bioactive compounds, use, and clinical trials. Int. J. Mol. Sci..

[15] Kawagishi H., Shimada A., Shirai R., Okamoto K., Ojima F., Sakamoto H., Ishiguro Y., Furukawa S. (1994). Erinacines A, B and C, strong stimulators of nerve growth factor (NGF)-synthesis, from the mycelia of *Hericium erinaceum*. Tetrahedron Lett..

[16] Wang K., Bao L., Qi Q., Zhao F., Ma K., Pei Y., Liu H. (2015). Erinacerins C–L, Isoindolin-1-ones with *α*-Glucosidase Inhibitory Activity from Cultures of the Medicinal Mushroom Hericium erinaceus. J. Nat. Prod..

[17] Thongbai B., Rapior S., Hyde K.D., Wittstein K., Stadler M. (2015). *Hericium erinaceus*, an amazing medicinal mushroom. Mycol. Prog..

[18] Li I.C., Lee L.Y., Tzeng T.T., Chen W.P., Chen Y.P., Shiao Y.J., Chen C.C. (2018). Neurohealth Properties of *Hericium erinaceus* Mycelia Enriched with Erinacines. Behav. Neurol..

[19] Chiu C.H., Chyau C.C., Chen C.C., Lee L.Y., Chen W.P., Liu J.L., Lin W.H., Mong M.C. (2018). Erinacine A-Enriched *Hericium erinaceus* Mycelium Produces Antidepressant-Like Effects through Modulating BDNF/PI3K/Akt/GSK-3*β* Signaling in Mice. Int. J. Mol. Sci..

[20] Lee S.L., Hsu J.Y., Chen T.C., Huang C.C., Wu T.Y., Chin T.Y. (2022). Erinacine A Prevents Lipopolysaccharide-Mediated Glial Cell Activation to Protect Dopaminergic Neurons against Inflammatory Factor-Induced Cell Death In Vitro and In Vivo. Int. J. Mol. Sci..

[21] Wang L.Y., Huang C.S., Chen Y.H., Chen C.C., Chen C.C., Chuang C.H. (2019). Anti-Inflammatory Effect of Erinacine C on NO Production Through Down-Regulation of NF-*κ*B and Activation of Nrf2-Mediated HO-1 in BV2 Microglial Cells Treated with LPS. Molecules.

[22] Lee K.F., Chen J.H., Teng C.C., Shen C.H., Hsieh M.C., Lu C.C., Lee K.C., Lee L.Y., Chen W.P., Chen C.C. (2014). Protective Effects of *Hericium erinaceus* Mycelium and Its Isolated Erinacine A against Ischemia-Injury-Induced Neuronal Cell Death via the Inhibition of iNOS/p38 MAPK and Nitrotyrosine. Int. J. Mol. Sci..

[23] Tsai P.C., Wu Y.K., Hu J.H., Li I.C., Lin T.W., Chen C.C., Kuo C.F. (2021). Preclinical Bioavailability, Tissue Distribution, and Protein Binding of Erinacine A in Rats. Molecules.

[24] Hu J.H., Li I.C., Lin T.W., Chen W.P., Lee L.Y., Chen C.C., Kuo C.F. (2019). Absolute Bioavailability, Tissue Distribution, and Excretion of Erinacine S in Rats. Molecules.

[25] Wei J., Li J., Feng X., Zhang Y., Hu X., Hui H., Xue X., Qi J. (2023). Unprecedented neoverrucosane and cyathane diterpenoids with anti-neuroinflammatory activity from cultures of the culinary-medicinal mushroom *Hericium erinaceus*. Molecules.

[26] Hämäläinen M., Nieminen R., Vuorela P., Heinonen M., Moilanen E. (2007). Anti-inflammatory effects of flavonoids: Genistein, kaempferol, quercetin, and daidzein inhibit STAT-1 and NF-*κ*B activations, whereas flavone, isorhamnetin, naringenin, and pelargonidin inhibit only NF-*κ*B activation along with their inhibitory effect on iNOS expression and NO production in activated macrophages. Mediat. Inflamm..

[27] Chen J.C., Ho F.M., Chao P.D.L., Chen C.P., Jeng K.C.G., Hsu H.B., Lee S.T., Wu W.T., Lin W.W. (2005). Inhibition of iNOS gene expression by quercetin is mediated by the inhibition of I*κ*B kinase, nuclear factor-kappa B and STAT1, and depends on heme oxygenase-1 induction in mouse BV-2 microglia. Eur. J. Pharmacol..

[28] Ferreira L.G., Dos Santos R.N., Oliva G., Andricopulo A.D. (2015). Molecular docking and structure-based drug design strategies. Molecules.

[29] Salmaso V., Moro S. (2018). Bridging molecular docking to molecular dynamics in exploring ligand-protein recognition process: An overview. Front. Pharmacol..

[30] Sadybekov A.V., Katritch V. (2023). Computational approaches streamlining drug discovery. Nature.

[31] Mattace Raso G., Meli R., Di Carlo G., Pacilio M., Di Carlo R. (2001). Inhibition of inducible nitric oxide synthase and cyclooxygenase-2 expression by flavonoids in macrophage J774A.1. Life Sci..

[32] Mukherjee S., Balius T.E., Rizzo R.C. (2010). Docking Validation Resources: Protein Family and Ligand Flexibility Experiments. J. Chem. Inf. Model..

[33] Lin J.Y., Chen Y.P., Lin T.W., Li T.J., Chen Y.W., Li I.C., Chen C.C. (2024). Discovery of a New Compound, Erinacerin W, from the Mycelia of *Hericium erinaceus*, with Immunomodulatory and Neuroprotective Effects. Molecules.

[34] Deshmukh S., Dufossé L., Chhipa H., Saxena S., Mahajan G., Gupta M. (2021). *Hericium erinaceus*—A rich source of diverse bioactive metabolites. Fungal Biotec.

[35] Qiu Y., Lin G., Liu W., Zhang F., Linhardt R., Wang X., Zhang A. (2023). Bioactive compounds in *Hericium erinaceus* and their biological properties: A review. Food Sci. Hum. Wellness.

[36] Chaudhary M., Tyagi K. (2024). A review on molecular docking and its application. Int. J. Adv. Res..

[37] Pantsar T., Poso A. (2018). Binding Affinity via Docking: Fact and Fiction. Molecules.

[38] Hou T., Wang J., Li Y., Wang W. (2011). Assessing the Performance of the MM/PBSA and MM/GBSA Methods. 1. The Accuracy of Binding Free Energy Calculations Based on Molecular Dynamics Simulations. J. Chem. Inf. Model..

[39] Garcin E.D., Arvai A.S., Rosenfeld R.J., Kroeger M.D., Crane B.R., Andersson G., Andrews G., Hamley P.J., Mallinder P.R., Nicholls D.J. (2008). Anchored plasticity opens doors for selective inhibitor design in nitric oxide synthase. Nat. Chem. Biol..

[40] Pettersen E., Goddard T., Huang C., Couch G., Greenblatt D., Meng E., Ferrin T. (2004). UCSF Chimera—A visualization system for exploratory research and analysis. J. Comput. Chem..

[41] Daina A., Michielin O., Zoete V. (2017). SwissADME: A free web tool to evaluate pharmacokinetics, drug-likeness and medicinal chemistry friendliness of small molecules. Sci. Rep..

[42] Sander T., Freyss J., von Korff M., Rufener C. (2015). DataWarrior: An Open-Source Program For Chemistry Aware Data Visualization And Analysis. J. Chem. Inf. Model..

[43] Lipinski C., Lombardo F., Dominy B., Feeney P. (2001). Experimental and computational approaches to estimate solubility and permeability in drug discovery and development settings. Adv. Drug Deliv. Rev..

[44] Eberhardt J., Santos-Martins D., Tillack A.F., Forli S. (2021). AutoDock Vina 1.2.0: New Docking Methods, Expanded Force Field, and Python Bindings. J. Chem. Inf. Model..

[45] Morris G.M., Huey R., Lindstrom W., Sanner M.F., Belew R.K., Goodsell D.S., Olson A.J. (2009). AutoDock4 and AutoDockTools4: Automated docking with selective receptor flexibility. J. Comput. Chem..

[46] Abraham M., Murtola T., Schulz R., Páll S., Smith J., Hess B., Lindahl E. (2015). GROMACS: High performance molecular simulations through multi-level parallelism from laptops to supercomputers. SoftwareX.

[47] Huang J., Rauscher S., Nawrocki G., Ran T., Feig M., de Groot B., Grubmüller H., MacKerell A.J. (2017). CHARMM36m: An improved force field for folded and intrinsically disordered proteins. Nat. Methods.

[48] Jorgensen W., Chandrasekhar J., Madura J., Impey R., Klein M. (1983). Comparison of simple potential functions for simulating liquid water. J. Chem. Phys..

[49] Bussi G., Donadio D., Parrinello M. (2007). Canonical sampling through velocity rescaling. J. Chem. Phys..

[50] Parrinello M., Rahman A. (1981). Polymorphic transitions in single crystals: A new molecular dynamics method. J. Appl. Phys..

[51] Hess B., Bekker H., Berendsen H., Fraaije J. (1997). LINCS: A linear constraint solver for molecular simulations. J. Comput. Chem..

[52] Darden T., York D., Pedersen L. (1993). Particle mesh Ewald: An N·log(N) method for Ewald sums in large systems. J. Chem. Phys..

[53] Valdés-Tresanco M., Valdés-Tresanco M., Valiente P., Moreno E. (2021). gmxMMPBSA: A new tool to perform end-state free energy calculations with GROMACS. J. Chem. Theory Comput..

[54] R Core Team (2024). R: A Language and Environment for Statistical Computing.

[55] Wickham H. (2016). ggplot2: Elegant Graphics for Data Analysis.

